# Nd:YAG-photobiomodulation enhanced ADSCs multilineage differentiation and immunomodulation potentials

**DOI:** 10.1007/s10103-023-03818-x

**Published:** 2023-08-22

**Authors:** Linhai He, Yi Zheng, Meng Liu, Xian Dong, Lihang Shen, Yang He, Jingang An, Yi Zhang

**Affiliations:** 1 First Clinical Division, Peking University School and Hospital of Stomatology & National Center for Stomatology & National Clinical Research Center for Oral Diseases & National Engineering Research Center of Oral Biomaterials and Digital Medical Devices, Beijing, People’s Republic of China; 2 Department of Oral and Maxillofacial Surgery, Peking University School and Hospital of Stomatology & National Center for Stomatology & National Clinical Research Center for Oral Diseases & National Engineering Research Center of Oral Biomaterials and Digital Medical Devices, 22 Zhongguancun Nandajie, Haidian District, Beijing, 100081 People’s Republic of China; 3 Laser and Cosmetic Surgery Division, Peking University School and Hospital of Stomatology & National Center for Stomatology & National Clinical Research Center for Oral Diseases & National Engineering Research Center of Oral Biomaterials and Digital Medical Devices, Beijing, People’s Republic of China

**Keywords:** Photobiomodulation, Adipose tissue-derived stem cells, Immunomodulation potentials, Multilineage differentiation

## Abstract

To investigate the effects of Nd: YAG (1064 nm) photobiomodulation on multilineage differentiation and immunomodulation potentials of adipose tissue-derived stem cells (ADSCs) in vitro and in vivo. For in vitro experiments, cells were divided into the control group (non-irradiated control ADSCs) and photobiomodulation groups. 0.5 J/cm^2^, 1 J/cm^2^, 2 J/cm^2^, and 4 J/cm^2^ were used for proliferation assays; for ADSCs adipogenic differentiation assays, 0.5 J/cm^2^, 1 J/cm^2^ were applied; 1 J/cm^2^ was used for migration and immunomodulation assays. The differentiation abilities were assessed by qPCR, Oil Red O staining, and Alizarin Red staining. The immunomodulation potential was assessed by qPCR and human cytokine array. DSS-induced colitis model. was used to test the effect of photobiomodulation on ADSCs immunomodulation potentials in vivo. Nd:YAG-based photobiomodulation dose-dependently promoted ADSCs proliferation and migration; 1 J/cm^2^ showed the best promotion effect on proliferation. Moreover, Nd:YAG photobiomodulation promoted ADSCs osteogenic differentiation and brown adipose adipogenic differentiation. The potential immunomodulation assays showed Nd:YAG photobiomodulation improved Anti-inflammation capacity of ADSCs and photobiomodulation irradiated ADSCs effectively alleviated DSS-induced colitis severity in vivo. Our study suggests Nd:YAG photobiomodulation might enhance the ADSCs multilineage differentiation and immunomodulation potentials. These results might help to enhance ADSCs therapeutic effects for clinical application. However, further studies are needed to explore the mechanisms of Nd:YAG photobiomodulation promoting multilineage differentiation and immunomodulation potentials of ADSCs.

## Introduction

Mesenchymal stem cells (MSCs) are a type of stromal stem cells that can self-renew, exhibit multilineage differentiation, and have immunomodulation potentials. MSCs have been widely used in tissue regeneration engineering and for treating autoimmune diseases and wound healing [1–3]. Adipose tissue-derived stem cells (ADSCs), an important source of MSCs, are isolated from adipose tissue. Compared to other sources of MSCs, ADSCs can be easily obtained, are associated with less morbidity to donors, are less immunogenic, and are more genetically stable in long-term culture, which makes these cells a promising source of stem cell therapies for clinical approaches [4, 5]. ADSCs can differentiate into different types of cells, which then secrete paracrine factors or regulatory mediators of immune function. ADSCs-derived cell therapy has been used to treat autoimmune diseases, neurological diseases, and skeletal and soft tissue diseases, such as SLE (systemic lupus erythematosus), Type 1 diabetes mellitus, osteoporosis, bone nonunion, and skin wounds [6–9]. Furthermore, our previous study showed that ADSCs effectively alleviates the symptoms and reduce pancreatic islets' inflammation in type 1 diabetes mellitus mice [10]. Moreover, our recent study suggested that ADSCs prevent medication-related osteonecrosis of the jaw (MRONJ) by promoting gingival healing and bone remodeling [11, 12]. However, many ADSCs are often needed for stem cell therapies, which require advanced cell expansion technologies; in addition, the delivery efficiency and regenerative functions of these cells (including differentiation, viability, and paracrine ability) for tissue repair and regeneration need to be strengthened [10, 13, 14].

Low-level laser therapy (LLLT), also known as photobiomodulation (PBM) therapy, is a non-invasive cell activator that induces athermic, nondestructive photobiological processes, and can accelerate wound healing by inducing or inhibiting the signaling associated with the activation of growth factors and cellular metabolism [15–19]. Some studies have suggested that photobiomodulation can enhance stem cell proliferation and differentiation and prevent stem cell apoptosis [20–24]. Nd:YAG lasers, working at a wavelength of 1064 nm, have higher tissue penetration compared with diode lasers [25] and are widely used in soft or deeper tissue applications. However, the effect of Nd:YAG-based photobiomodulation on ADSCs capacities, especially on the immunomodulation potentials, has not been reported. Thus, in this study, we assess the effects of Nd: YAG-based LLLT on ADSCs capacities and immunomodulation potential by investigating cell proliferation, multi-differentiation, and therapeutic potential in DSS (dextransulfatesodium)-induced colitis models in vivo.

## Methods and materials

### ADSCs culture

ADSCs were provided by ADSCs bank, as described in our previous study [11, 12]. ADSCs were cultured in alpha-MEM medium (Gibco, Grand Island, NY, USA) supplemented with 10%FBS and 1%Penicillin/Streptomycin in a humidified atmosphere containing 5%CO_2_/95% air at 37ºC. The cells were passaged 3 times before starting the experiments.

### Cell proliferation assays

Cell Counting Kit-8 (CCK-8, Dojindo Laboratories, Japan) was used to assess cell proliferation. The third-passage ADSCs cells were seeded at a density of 2,000 cells per well in 96-well plates for 1, 3, 5, 7, 9, 11, and 13 days. At each time point, 20 μl of sterile CCK-8 reagent was added to each well and incubated for 2 h at 37 °C in cell incubator. Then the relative cell number was assessed by 450 nm optical density (od) values according to the manufacturer’s instructions.

### Adipogenic and osteogenic differentiation assays

For adipogenic differentiation, cells were cultured in an adipogenic culture medium supplemented with 0.5 µm hydrocortisone, 0.5 mm 3-isobutyl-methylxanthine, 10 µg/mL insulin, 60 µm indomethacin (Sigma-Aldrich, USA), and 10%FBS. The adipogenic medium was changed every 2 days. After 7 d, mRNA was isolated for qPCR analysis. After 21 d, the cells were stained for cellular lipiddroplets with Oil Red O (Sigma-Aldrich, USA).

For osteogenic differentiation, cells were cultured in an osteogenic medium supplemented with 10 nM dexamethasone, 0.1 mm L-ascorbicacid phosphate, and 10 mM β-glycerophosphate (Sigma-Aldrich, USA). The osteogenic medium was changed every 2 days. After 7 d, mRNA was isolated for qPCR analysis. After 21 d, calcium nodes were stained with 2% Alizarin red (Sigma-Aldrich, USA).

### Cell migration assays

Cells were seeded into the Culture-Insert 3 ibidiR chamber (ibidi GmbH, Gräfelfing, Germany) following the manufacturer’s instructions. 5,000 cells/chamber were seeded at cell culture plates and cultured for 12 h in cell incubator. Then the ADSCs medium was replaced with ADSCs migration medium. Subsequently, the silicone insert devices were carefully removed, and 500 μm-width cell-free gaps were created. Photographs were taken at 0 and 24 h after insert devices were removed, and the average size of the gaps was quantitated and calculated by Image J software (National Institutes of Health).

### qPCR assays

Total RNA was isolated using TRIzol reagent (Invitrogen Life Technologies, USA). Complementary DNAs (cDNAs) were prepared using the GoScript Reverse Transcription System (Promega, Promega Corporation, USA). Real-time polymerase chain reaction was performed with an ABI Prism 7500 (Thermo Fisher Scientific, USA). β-Actin was used to normalize gene expression, and the relative mRNA expression levels were calculated. Primers used in this study are shown in Table 1.

**Table 1:** Primers used in real-time polymerase chain reaction gene expression analysis

Genes			Primers sequences
β-actin			
	forward		CATGTACGTTGCTATCCAGGC
	reverse		CTCCTTAATGTCACGCACGAT
Runx2			
	forward		TGGTTACTGTCATGGCGGGTA
	reverse		TCTCAGATCGTTGAACCTTGCTA
ALP			
	forward		AACATCAGGGACATTGACGTG
	reverse		GTATCTCGGTTTGAAGCTCTTCC
OCN			
	forward		GGCGCTACCTGTATCAATGG
	reverse		GTGGTCAGCCAACTCGTCA
PPAR-γ2			
	forward		GGGATCAGCTCCGTGGATCT
	reverse		TGCACTTTGGTACTCTTGAAGTT
LPL			
	forward		TCATTCCCGGAGTAGCAGAGT
	reverse		GGCCACAAGTTTTGGCACC
UCP1			
	forward		TCTCTCAGGATCGGCCTCTA
	reverse		GTGGGTTGCCCAATGAATAC
PRDM16			
	forward		CTTCGGATGGGAGCAAATACTG
	reverse		TCCACGCAGAACTTCTCACTG
TNFRSF9			
	Forward		AGCTGTTACAACATAGTAGCCAC
	reverse		GGACAGGGACTGCAAATCTGAT

### Cytokine array and cytokine-gene expression analysis

Culture supernatants and mRNAs from ADSCs treated by LPS (Lipopolysaccharide) (100 ug/ml) for 1 h were collected and analyzed using Cytokine Array Panel Array Kit (R&D Systems, USA). qPCR was then performed according to the manufacturer’s instructions. The results were scanned and analyzed to calculate blot intensity.

### ADSCs transplantation into acute colitis mice

Balb/c male nude mice, 6–8 weeks old, weighing 20–25 g, were obtained from Vital River Laboratories, China. All the animals were housed in an environment with a temperature of 22 ± 1 ºC, relative humidity of 50 ± 1%, and a light/dark cycle of 12/12 h. All animal studies (including the mice euthanasia procedure) were done in compliance with the regulations and guidelines of Peking University institutional animal care and conducted according to the AAALAC and the IACUC guidelines. Also, the study was approved by The Ethics Committee of the Peking University Health Science Center (LA2018265).

The mice were randomly divided into 4 groups (*n* = 6), negative control group, PBS group, control ADSCs group and photobiomodulation group. Negative control group were healthy mice. Acute colitis was induced by administering 3% (w/v) DSS (molecular mass: 36,000–50,000 Da; MP Biochemicals) through drinking water for 8 days. Acute colitis mice were then divided into three groups (*n* = 6/group): PBS (Phosphate Buffered Saline) group, ADSCs group and LLLT irradiated ADSCs group. ADSCs group and photobiomodulation irradiated ADSCs group were intravenously infused (1.0 × 10^6^ cells) into the colitis mice 3 days post-DSS induction. All mice were harvested at 8 days post-DSS induction for further analysis.

### Colitis activity index

Cooper’s grading system for the degree of colonic inflammation was used, and the disease activity index (DAI) was determined for DSS-induced mice as previously described [26]. The DAI scoring criteria included occult/gross rectal bleeding, stool consistency, and weight loss. Each DAI parameter was scored between 0 (undamaged) and 4 (severe damage), and the arithmetic average was taken.

- Score 0, None weight loss, normal stool, negative Occult
- Score 1, 1%-5% weight loss, Loose stools, negative Occult
- Score 2, 5%-10% weight loss, loose stools, Hemoccult positive
- Score 3, 10%-20% weight loss, Diarrhea, Hemoccult positive
- Score 4, > 20% weight loss, Diarrhea, gross bleeding

### Laser irradiation

An Nd:YAG laser (FOTONA, Slovenia) with a wavelength of 1064 nm was used. MarcCO handpieces was used for ADSCs photobiomodulation. The operating mode was set pulsed, the laser pulse duration was 0.1 s (100 ms), and the frequency was 1 Hz. The laser spot diameter was set 43 mm. The average laser power density was set as 0.5 w/cm^2^ and the laser beam was irradiated vertically on ADSCs. The average energy density for ADSCs proliferation assays was set and operated as 0.5 J/cm^2^ (irradiation 1 s at average laser power density 0.5 w/cm^2^), 1 J/cm^2^ (irradiation 2 s at average laser power density 0.5 w/cm^2^), 2 J/cm^2^ (irradiation 4 s at average laser power density 0.5 w/cm^2^), and 4 J/cm^2^ (irradiation 8 s at average laser power density 0.5 w/cm^2^). The average energy density for ADSCs adipogenic differentiation assays was set and operated as 0.5 J/cm^2^ (irradiation 1 s at average laser power density 0.5 w/cm^2^), 1 J/cm^2^ (irradiation 2 s at average laser power density 0.5 w/cm^2^). The average energy density for ADSCs migration and immunomodulation ability assays was set and operated as 1 J/cm^2^ (irradiation 2 s at average laser power density 0.5 w/cm^2^). Non-irradiated control ADSCs were kept under the same conditions. The laser irradiation was conducted every two days until cell collection.

### Statistical analysis

Independent two-tailed Student’s t-tests analyzed comparisons between two groups, and comparisons between more than two groups were analyzed by one-way ANOVA. P values < 0.05 were considered statistically significant.

## Result

### Effect of photobiomodulation on proliferation and migration abilities of ADSCs

**Fig. 1:**
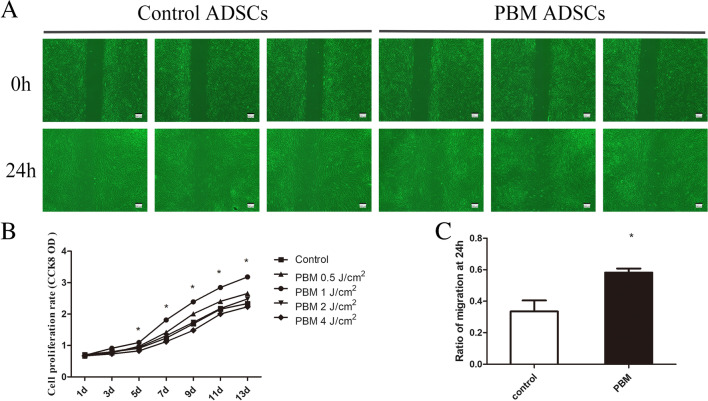
PBM promotes ADSCs migration and proliferation. **A** The migration assay of ADSCs treated with PBM. **B** Comparison of proliferative rates of ADSCs by CCK8. **(C)** Quantification of the migration rate at 24 h. *p < 0.05

Proliferation and migration abilities are the fundamental properties of ADSCs during wound healing. The CCK8 results showed that the power density of photobiomodulation exhibited a biphasic effect on ADSCs proliferation ability, and the power density of photobiomodulation at 1 J/cm^2^ had the best-irradiated effects on ADSCs proliferation (13d, control vs PBM 1 J/cm^2^, 2.336 ± 0.140 vs 3.180 ± 0.112, *p* = 0.0079) (Fig. 1B). In addition, the migration assay result also showed that the power density of photobiomodulation at 1 J/cm^2^ effectively increased ADSCs migration ability (control vs PBM, 0.3358 ± 0.1386 vs 0.5820 ± 0.0517, *p* = 0.0286) (Fig. 1A and C).

### Effects of photobiomodulation on adipogenic and osteogenic differentiation of ADSCs

**Fig. 2:**
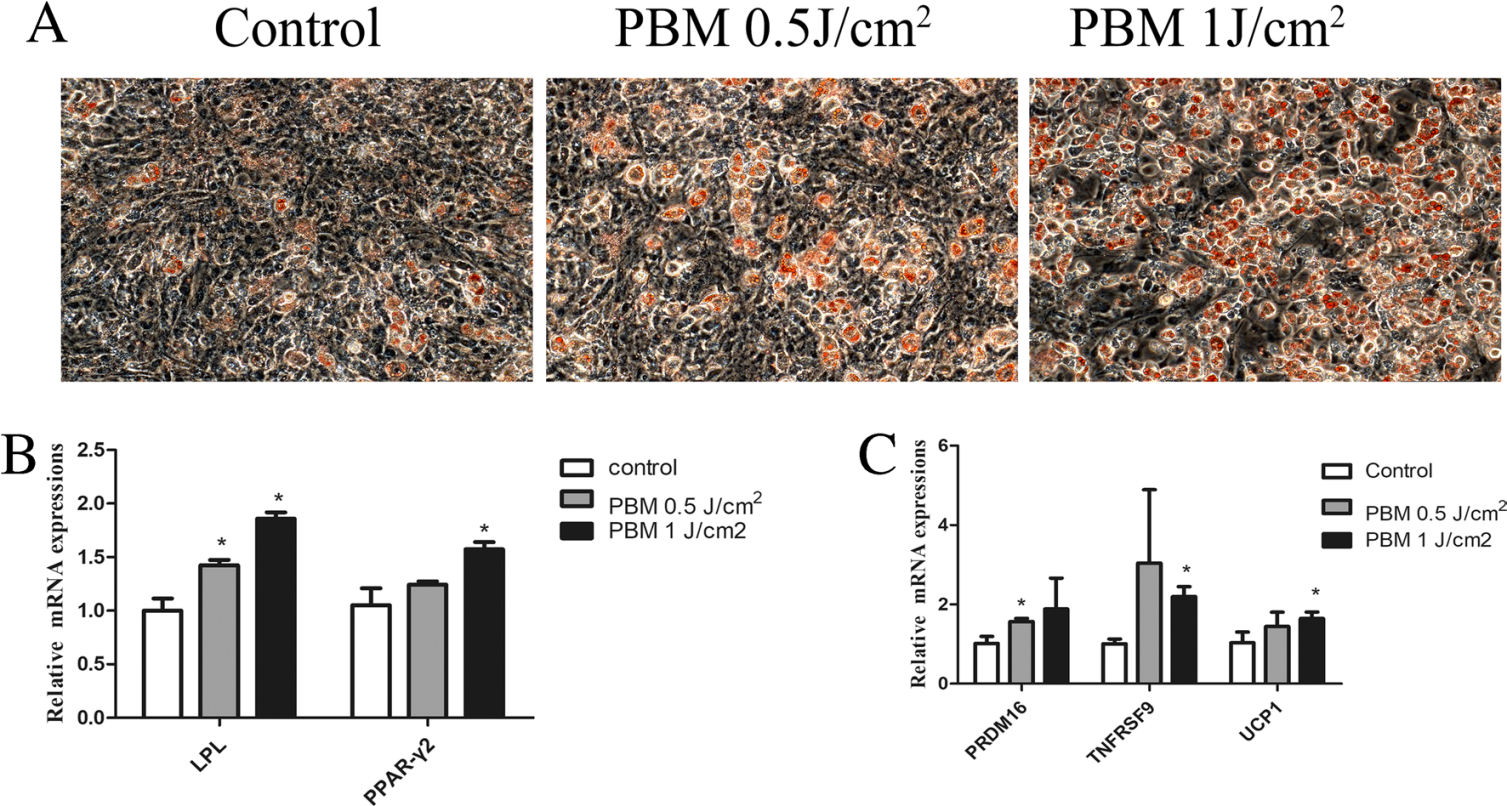
PBM promotes adipogenic differentiation of ADSCs toward brown adipose tissue. **A** Adipogenic inducing for 21 d and staining by Oil Red O staining. **B** mRNA expressions of peroxisome proliferator-activated receptor (PPAR)–γ2 and lipoprotein lipase (LPL) by qPCR (**C**) mRNA expressions of brown adipose differentiation marker. *p < 0.05

The Oil Red O assays and qPCR were used to assess the effects of photobiomodulation on adipogenic differentiation. photobiomodulation at 1 J/cm^2^ promoted adipogenic differentiation by up-regulating PPAR-γ2 (peroxisome proliferator-activated receptor-gamma2) (control vs PBM 1 J/cm^2^, 1.04 ± 0.221 vs 1.565 ± 0.069, *p* = 0.0025) and LPL (lipoprotein lipase) (control vs PBM 1 J/cm^2^, 0.995 ± 0.196 vs 1.841 ± 0.061, *p* = 0.0159) gene expressions (Fig. 2A and B). In addition, photobiomodulation irradiated ADSCs expressed higher brown adipose differentiation markers, such as UCP1 (uncoupling protein 1) (control vs PBM 1 J/cm^2^, 1.028 ± 0.323 vs 1.65 ± 0.182, *p* = 0.0043) and PRDM16 (PR domain-containing protein 16) (control vs PBM 0.5 J/cm^2^, 1.11 ± 0.316 vs 1.556 ± 1.174, *p* = 0.0317) (Fig. 2C).

**Fig. 3:**
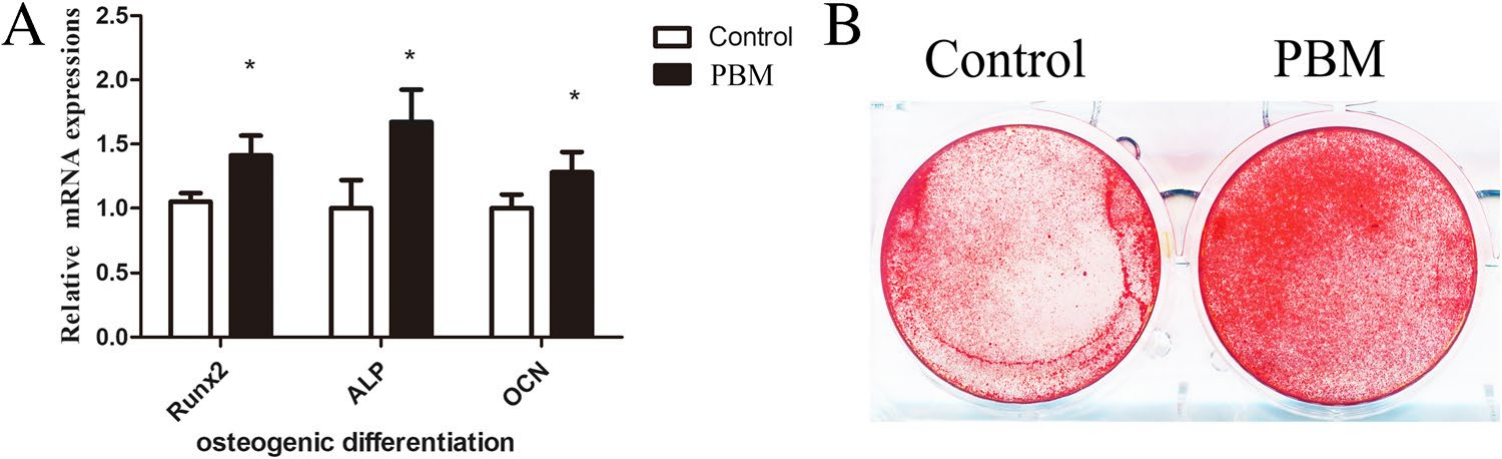
PBM promotes the osteogenic differentiation of ADSCs. **A** mRNA expression levels of Runx2, alkaline phosphatase (ALP), and osteocalcin (OCN) determined by PCR. **B** Alizarin red staining for i*n vitro* calcium deposition

The qPCR and Alizarin Red staining were then used to assess the effect of photobiomodulation on the osteogenic differentiation of ADSCs. The qPCR results showed that Runx2 (Runt-related transcription factor 2) (control vs PBM 1 J/cm^2^, 1.060 ± 0.119 vs 1.442 ± 0.237, *p* = 0.0234), ALP (alkaline phosphatase) (control vs PBM 1 J/cm^2^, 1.025 ± 0.219 vs 1.649 ± 0.251, *p* = 0.0135), and OCN (osteocalcin) (control vs PBM 1 J/cm^2^, 1.01 ± 0.108 vs 1.291 ± 0.152, *p* = 0.046) mRNA expressions were higher in photobiomodulation irradiated ADSCs during osteogenic induction (Fig. 3A). Consistently, the Alizarin Red staining assay photobiomodulation irradiated ADSCs formed more calcium depositions than the control group when during ADSCs osteogenic induction (Fig. 3B).

### Effect of photobiomodulation on the therapeutic potential of ADSCs in DSS-induced colitis model

**Fig. 4:**
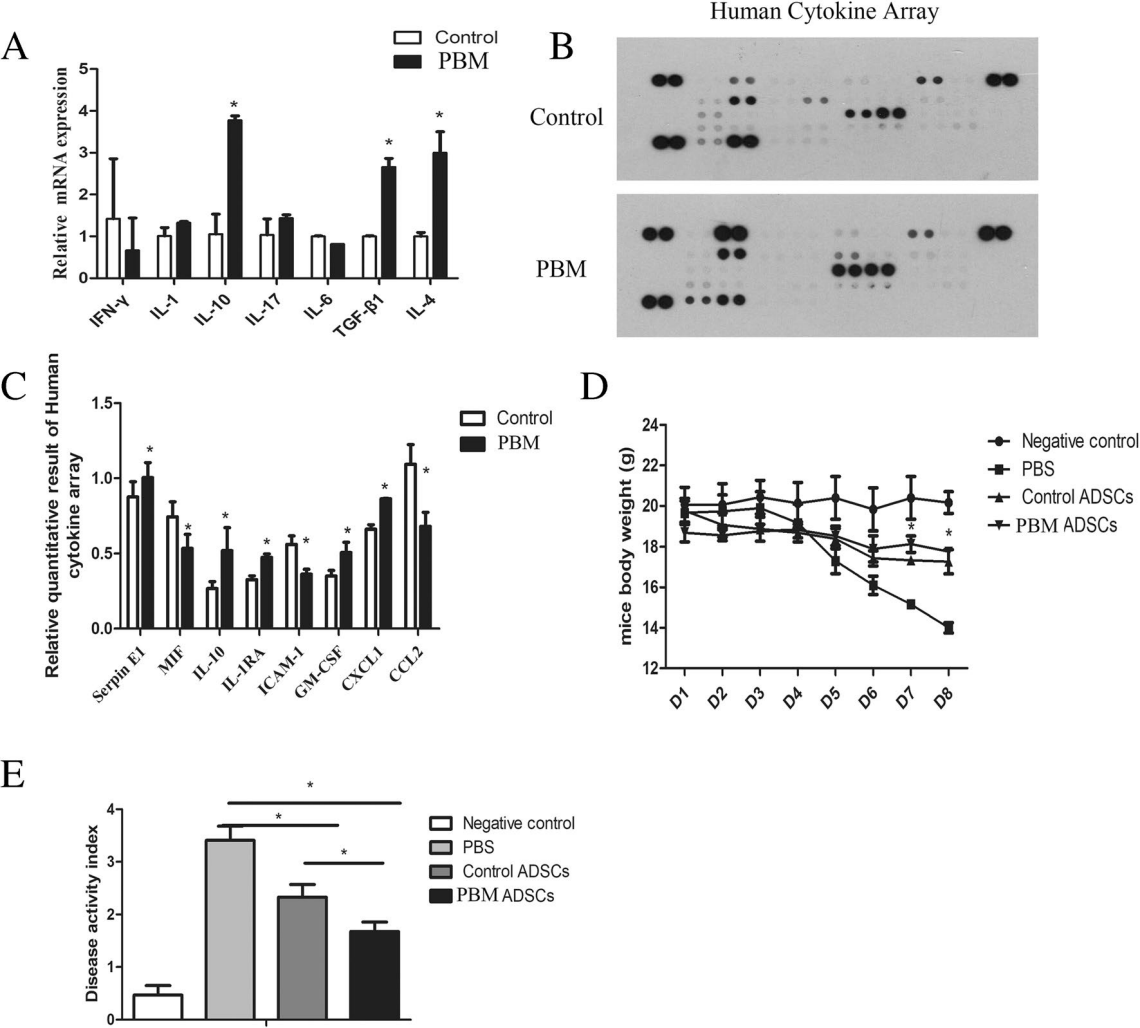
PBM enhances the immunomodulation potentials of ADSCs. **A** mRNA expressions of inflammation cytokines markers. **B**, **C** Cytokine array results shows that PBM increases anti-inflammation cytokines expressions. (**D**) PBM-radiated ADSCs rescued more weight loss in DSS-induced colitis mice than control ADSCs. **E** The disease activity index shows that PBM-irradiated ADSCs decrease the disease activity more than control ADSCs. *p < 0.05

To explore the effect of photobiomodulation on immunomodulation potential of ADSCs, the qPCR and protein cytokine array was used to assess the cytokines level of ADSCs. The results showed anti-inflammation cytokines IL-4 (control vs PBM 1 J/cm^2^, 0.9825 ± 0.2047 vs 3.133 ± 0.7524, *p* = 0.0117), IL-10 (control vs PBM 1 J/cm^2^, 1.103 ± 0.589 vs 3.708 ± 0.188, *p* = 0.0035), and IL-1RA (control vs PBM 1 J/cm^2^, 0.323 ± 0.035 vs 0.475 ± 0.028, *p* = 0.0011) were increased, while IL-6 (control vs PBM 1 J/cm^2^, 1.023 ± 0.175 vs 0.817 ± 0.058, *p* = 0.113) expression down-regulated in photobiomodulation irradiated group vs. control group (Fig. 4A-C).

The in vivo assays showed there was significant weight loss in the PBS group of DSS-induced colitis, while ADSCs treatment effectively reduced weight loss and photobiomodulation irradiated ADSCs exhibited better therapeutic potential than non-irradiated ADSCs (DAI score, control ADSCs vs PBM ADSCs, 2.330 ± 0.335 vs 1.675 ± 0.251, *p* = 0.026) (Fig. 4D). Furthermore, the disease activity and severity of colitis were decreased more remarkably in photobiomodulation irradiated ADSCs group than in non-irradiated ADSCs group.

## Discussion

ADSCs-based cell therapies have drawn much attention in the clinical and scientific fields due to ADSCs' multi-differentiation and immunomodulation potential. Previous studies found that photobiomodulation at a wavelength of 635 nm significantly promotes ADSCs proliferation [27]. Our results showed that Nd:YAG-based photobiomodulation might effectively promote ADSCs proliferation in a dose-dependent manner; yet a very higher power density (2 J/cm^2^ and 4 J/cm^2^) suppressed ADSCs proliferation (Fig. 1B). These results suggest photobiomodulation might have a biphasic effect on ADSCs viability. Consistently, Liao et al*.* found 650 nm laser-based photobiomodulation at power density 4 J/cm^2^ promotes ADSCs proliferation compared to power density 8 J/cm^2^ [28]. Moreover, de Andrade et al*.* also reported 660 nm laser-based photobiomodulation promoted ADSCs proliferation at a power density of 0.5 J/cm^2^ and 2 J/ cm^2^, while higher energy at 5 J/cm^2^ impaired ADSCs proliferation ability [29]. However, the mechanisms are still unclear.

Karu et al*.* hypothesized that photoreceptor damage increases with laser energy dose, reducing the biomodulatory effect and inhibiting cell metabolism [30]. ADSCs migration toward the wound edge is one of the key processes in MSCs-facilitating wound healing. Therefore, enhancing the migration ability of ADSCs would benefit therapeutic strategies in wound healing. Our results showed that Nd: YAG-based photobiomodulation dramatically enhances ADSCs migration ability; this data are consistent with Yuji Tsuka et al*.* [31]. Moreover, Yin et al*.* demonstrated that photobiomodulation 660 nm stimulates ADSCs migration by activating MAPK/ERK signaling pathway and an anti-ERK agent (FR180204), significantly reducing the photobiomodulation-induced migration acceleration effect on ADSCs [32]. Thus, these results suggested Nd:YAG-based photobiomodulation promotes proliferation and migration abilities.

Multilineage-differentiation capacity is an important feature of ADSCs. Osteogenic differentiation of ADSCs has always been a topic in MSCs translational medicine. Previous studies have suggested that photobiomodulation may promote ADSCs osteogenic potential [33]. In this study, we found that Nd:YAG based photobiomodulation stimulates the osteogenic differentiation of ADSCs. Mechanistically, Dompe et al*.* found that photobiomodulation could increase intracellular Ca^2+^ and activate calcium-related signaling pathways [34]. Our previous study suggested that calcium signaling mediated by TRPM7 and TRPV1 exerts essential functions during MSCs osteogenic differentiation [35, 36]. Furtherly, Wang et al*.* found 420 nm and 540 nm photobiomodulation promote osteogenic differentiation of ADSCs (these effects were abrogated by TRPV1 and TRPC channel inhibitors). These results suggested calcium signaling might be one of the important factors in photobiomodulation promoting osteogenic differentiation of ADSCs.

Photobiomodulation has recently been widely applied in weight loss in clinical work [37, 38]. Thus, the effects of photobiomodulation on the adipogenic differentiation potential of ADSCs have drawn much attention. However, a previous study suggested 650 nm-photobiomodulation enhanced ADSCs adipogenic differentiation at 4 J/cm^2^ power density [28]. Consistently, our results also showed 1064 nm Nd:YAG photobiomodulation increases adipogenic genes expression and lipid droplets formation (Fig. 2). Interestingly, our results further showed Nd: YAG-based photobiomodulation increases UCP1, PRDM16, and TNFRSF9 expressions, which are markers of brown adipose tissue [39]. Accumulated studies have suggested that the plasticity of white adipose tissues and brown adipose tissues determines the individuals’ obese or lean, and UCP1-mediated activation of brown adipose tissue, which tends to be easier for thermogenesis and consumption, has been proposed a new treatment approach for combating obesity and its related diseases [40, 41]. Also, previous studies demonstrated that photobiomodulation increase mitochondrial ROS level in a dose-dependent manner [42], and mitochondrial ROS was reported to drive UCP1-meditated brown adipose tissue formation and sequent thermogenesis [43]. However, whether mitochondrial ROS was involved in photobiomodulation -promoted ADSCs brown adipose tissue differentiation is still not clear. In this study, though our results suggested Nd:YAG based photobiomodulation might promote ADSCs adipogenic differentiation potential towards brown adipose tissue, detailed mechanisms and in vivo studies need to be further explored.

Cytokines-mediated autocrine and paracrine effects are an important mechanism of ADSCs therapy [44]. Previous studies have suggested ADSCs as the important immunomodulator to suppress inflammatory responses by producing IL-10 [4]. Our previous studies have shown that medication-related osteonecrosis of the jaw may be prevented by ADSCs-produced TGF-β1 mediated gingival healing and bone remodeling [11, 12]. Photobiomodulation at 650 nm has been reported to increase VEGF, TGF-β, and PDGF expressions of ADSCs [28]. In our present study, Nd: YAG-based photobiomodulation increased IL-10, IL-4, IL-1RA, and TGF-β1 expressions and down-regulated CCL2 and MIF expressions (Fig. 4). These results suggested photobiomodulation might improve the immunomodulation potential by increasing anti-inflammation/pro-inflammation cytokines ratio. Consistently, Yin et al*.* reported 660 nm- photobiomodulation suppressed IL-6 and IL-8 expressions while increasing IL-4 and IL-10 expressions by suppressing phosphorylation of NF-κB in ADSCs in vitro [45]. To further explore the effects of photobiomodulation on the immunomodulation potential of ADSCs in vivo, the DSS-induced colitis model was used, and the results showed Nd:YAG-based photobiomodulation irradiated ADSCs exhibited better therapeutic effects in alleviating colitis by reducing weight loss and decreasing disease activity index of colitis compared with control ADSCs. However, the mechanism was not clear. Thus, in this study, our results suggested the Nd:YAG based photobiomodulation might enhance the immunomodulation potential of ADSCs by increasing anti-inflammation effects, and further study is needed to explore the mechanisms of photobiomodulation enhancing immunomodulation potential of ADSCs.

To sum up, this study demonstrated that Nd: YAG-based photobiomodulation promotes ADSCs proliferation, adipogenic and osteogenic differentiation capacities, increases anti-inflammation cytokines, and improves ADSCs immunomodulation potential. Thus, Nd:YAG based photobiomodulation might be an effective activator for ADSCs to enhance cell therapy. However, the mechanisms of Nd:YAG-based photobiomodulation enhancing ADSCs immunomodulation potential and promoting ADSCs towards brown adipose adipogenic differentiation are the main limitations of this study, which need to be further explored in the future.

## Data Availability

The data that supporting the finding of this study are available from the corresponding author upon reasonable request.
